# Swaging Plates

**Published:** 1853-10

**Authors:** 


*Swaging Plates.*—Dr. D. H. Taylor, of Newark, Ohio, gives the following as his method of swaging of plates: [See News-Letter, page 238.]

"My method is first to get the impression, and first metallic cast in the usual way; when I have obtained a correct metallic model, and before obtaining a counter model, I reduce to the consistence of paint, some Spanish whiting with water, in which I dissolve a small quantity of gum arabic. I then coat the first metallic cast with the above preparation to the thickness of the plate I wish to swage, after it has become hard and dry. I melt the tin, zinc, &c., and by pouring it over the prepared model, I obtain a counter model, which, when cold, I remove and wash the whiting from the first cast, and by placing the models together, space sufficient is left to admit the plate, which, by this method, can be swaged accurately; the plate also retains the same thickness throughont, and plates swaged in this manner are not liable to spring."

